# iGEM as a human iPS cell-based global epigenetic modulation detection assay provides throughput characterization of chemicals affecting DNA methylation

**DOI:** 10.1038/s41598-023-33729-4

**Published:** 2023-04-24

**Authors:** Satoshi Otsuka, Xian-Yang Qin, Wenlong Wang, Tomohiro Ito, Hiroko Nansai, Kuniya Abe, Wataru Fujibuchi, Yoichi Nakao, Hideko Sone

**Affiliations:** 1 Center for Health and Environmental Risk Research, National Institute for Environmental Studies, 16-2 Onogawa, Tsukuba, Ibaraki 305-8506 Japan; 2 Graduate School of Advanced Science and Engineering, Waseda University, 3-4-1 Okubo, Shinjuku-Ku, Tokyo, 169-8555 Japan; 3 Laboratory for Cellular Function Conversion Technology, RIKEN Center for Integrative Medical Sciences, 1-7-22 Suehiro, Tsurumi, Yokohama, Kanagawa 230-0045 Japan; 4 Department of Environmental Engineering, Graduate School of Engineering, Kyoto University, Kyoto University Katsura, Nishikyo-Ku, Kyoto, 615-8540 Japan; 5 Technology and Development Team for Mammalian Cellular Dynamics, BioResource Center, RIKEN Tsukuba Institute, 3-1-1 Koyadai, Tsukuba, Ibaraki 305-0074 Japan; 6 Center for iPS Cell Research and Application (CiRA), Kyoto University, 53 Kawahara-Cho, Sho-Goin, Sakyo-Ku, Kyoto, 606-8507 Japan; 7 Environmental Health and Prevention Research Unit, Department of Environmental Health and Preventive Medicine, Yokohama University of Pharmacy, 601 Matano, Totsuka, Yokohama 245-0066 Japan; 8 Present Address: Department of Cellular and Tissue Communication, Graduate School of Medicine and Faculty of Medicine, The University of Tokyo, 7-3-1 Hongo, Bunkyo-Ku, Tokyo, 113-8555 Japan

**Keywords:** Chemical biology, Developmental biology, Stem cells, Environmental sciences, Biomarkers, Diseases, Medical research, Risk factors

## Abstract

Chemical-induced dysregulation of DNA methylation during the fetal period is known to contribute to developmental disorders or increase the risk of certain diseases later in life. In this study, we developed an iGEM (iPS cell-based global epigenetic modulation) detection assay using human induced pluripotent stem (hiPS) cells that express a fluorescently labeled methyl-CpG-binding domain (MBD), which enables a high-throughput screening of epigenetic teratogens/mutagens. 135 chemicals with known cardiotoxicity and carcinogenicity were categorized according to the MBD signal intensity, which reflects the degree of nuclear spatial distribution/concentration of DNA methylation. Further biological characterization through machine-learning analysis that integrated genome-wide DNA methylation, gene expression profiling, and knowledge-based pathway analysis revealed that chemicals with hyperactive MBD signals strongly associated their effects on DNA methylation and expression of genes involved in cell cycle and development. These results demonstrated that our MBD-based integrated analytical system is a powerful framework for detecting epigenetic compounds and providing mechanism insights of pharmaceutical development for sustainable human health.

## Introduction

The early stage of life is controlled by significant changes in gene expression profiles, which are strictly regulated by epigenetic processes such as DNA methylation or histone acetylation^1^. In contrast, accumulation of epigenetic abnormalities during the fetal period may trigger reproductive or developmental toxicities^2^, as well as lifestyle-related diseases, such as diabetes, hypertension^3,4^, and neurodevelopmental disorders^5^. However, the contribution of chemical-induced dysregulation of DNA methylation during the early developmental stage is unclear. Dietary chemicals, such as folate, which is involved in C1 metabolism, and other natural compounds have a profound effect on health through interactions with the genome that alter gene expression and produce various consequences^3^. Coffee-associated changes in DNA methylation may explain the mechanism of action of coffee consumption and the reported risk of diseases in epigenome-wide association meta-analysis of DNA methylation^6^. Anti-cancer drugs have been shown to cause late-onset adverse effects such as cardiotoxicity and neuropathy via epigenetic changes including DNA methylation and histone deacetylation^7^. Pregnant women and fetuses are exposed to a wide range of substances due to lifestyle and occupational environment. As a result, there is an urgent need to comprehensively understand and quantitatively monitor the epigenetic influences of nutritional and environmental chemicals during the early stage of life. Abnormalities of DNA methylation, including DNA hypomethylation (decreased levels of DNA methylation) or hypermethylation (increased levels of DNA methylation), have been already reported in cancer and various other diseases^8,9^. In vitro cell models and mouse models have shown that various chemicals can cause changes in DNA methylation^10^. For instance, tris(1,3-dichloroisopropyl)phosphate, a flame retardant, reduces DNA methylation at the genome-wide level in early zebrafish embryos^11^. Tributyltin and triphenyltin, used as anti-fouling paints, induce DNA hypomethylation in *Sebastiscus marmoratus* liver^12^. Sodium arsenite induces global DNA hypomethylation in C3H mice with carcinogen-induced liver cancer^13^. In contrast, there is a lack of research on chemicals that induce DNA hypermethylation, and few chemicals toxic against DNA methylation have been evaluated. Therefore, there is a need to develop methods that can easily evaluate the effects of chemicals on global DNA methylation.

High throughput assays to evaluate epigenetic effects of chemicals have been developed, but most of them are limited to epigenetic enzyme-based assays, in which enzymatic activities of chemicals are evaluated^14–16^. Owing to the fact that the reaction between chemicals and enzymes does not always match the reaction in cells or in vivo, there is a need to develop an assay to evaluate the effects of chemicals under conditions in an environment that is as similar as possible to in vivo. For this reason, some researchers have aimed to develop methods to observe states of DNA methylation in living cells^17,18^. The methyl-CpG-binding domain (MBD) is a functional domain in proteins that binds to methylated CpG sites. Eleven proteins with an MBD (e.g., MBD1 and MeCP2) have been identified in mammals. The MBD suppresses gene expression by binding to methylated promoter regions and, together with other histone modifications, induces changes in the chromatin structure^19^. Previous studies using mouse embryonic stem (ES) cells transfected with a fusion gene consisting of the MBD, enhanced green fluorescent protein, and nuclear localization signal (nls) showed that the localization of the MBD protein in the nuclei changed depending on exposure to insecticides or the differentiation status of cells^20,21^. Another report showed MBD associated with global DNA methylation in ES cells or differentiated cells during early development^22^. The ten-eleven translocation protein-mediated DNA modification pathway plays an important role in regulating DNA methylation and demethylation homeostasis during heart development in the early embryonic stage^23^. In pluripotent cells, active status genomic 5-methyl-2′-deoxycytidine (mdC) is critical during turnover processes^24^. Therefore, induced pluripotent stem cells (iPSCs) and ES cells are considered highly sensitive to changes in chemical-induced DNA methylation. Our previous report showed the predictive toxicity of chemicals using human ES cells, which demonstrate higher sensitivity than other cells^25,26^. In addition, effects of chemicals on mCherry-MBD lesions respond to DNA methylation not only in the heterochromatin region, but also in the promoter/enhancer/gene center region^19^. These comprehensive phenotypic analyses thus promise to shed new light onto the relationship between epigenetic changes and heterochromatin dynamics. Our present study examined whether a fusion system consisting of the MBD and fluorescent protein in human iPSCs could be used to rapidly screen chemicals that cause alterations in nuclear spatial distribution/concentration of DNA methylation, in combination with genome-wide analyses of gene expression and DNA methylation, knowledge-based analyses, and chemical screening using a chemical library. Our findings showed that changes in fluorescent MBD foci reflect the effects of these chemicals on nuclear spatial distribution/concentration of DNA methylation in human iPSCs. Hypermethylation agents with constitutive androstane receptor (CAR) antagonistic or histone H2A histone family member X (H2AX) phosphorylation activities increased DNA methylation and downregulated the expression of genes, such as *JAG1* or *CCND2*, that are involved in cell development and cell cycle. These findings indicate that our high-throughput integrated analytical strategy may serve as a resource and framework for examining a large number of epigenetic compounds for DNA methylation, which may contribute to pharmaceutical development and comprehensive evaluation of the risk of complicated environmental exposures during the early stage of life.

## Results

### Establishment of an assay using mCherry-MBD-nls human iPSCs to evaluate the effects of chemicals on DNA methylation

**Figure 1:**
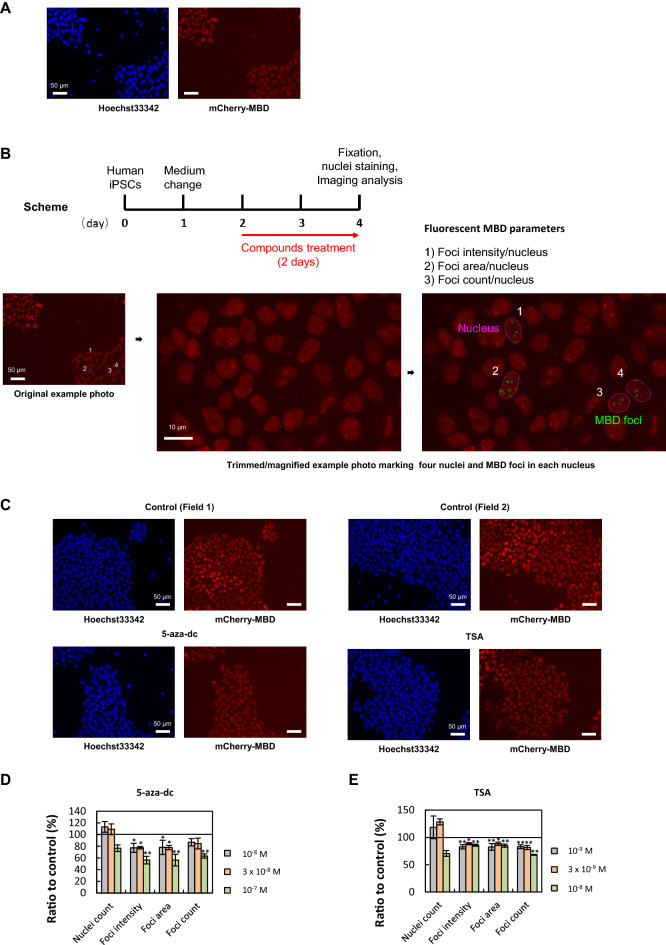
Establishment of an assay using mCherry–MBD–nls human iPSCs to evaluate the effects of chemicals on DNA methylation. (**A**) A fusion gene consisting of the methyl-CpG-binding domain (MBD), nuclear localization signal (nls), and red fluorescent protein, mCherry, was introduced into human iPSCs. Blue and red colors represent Hoeschst33342 and mCherry–MBD, respectively, in microscopic images of mCherry–MBD–nls human iPSC. (**B**) Scheme of the assay to evaluate the effects of chemicals on nuclear spatial distribution/concentration of DNA methylation using mCherry-MBD-nls human iPSCs. Fluorescent MBD parameters: foci (the green circle) count, intensity, and area in nucleus (the pink circle) were automatically calculated using IN Cell Analyzer 1000. Original and trimmed/magnified example photos of four nuclei (,which were picked up and selected) in (**A**) were shown. (**C**) Representative microscopic images of mCherry-MBD-nls human iPSCs. Images of cells treated with 0.1% DMSO (Control), 1 × 10^−7^ M 5-aza-2′-deoxycytidine (5-aza-dc), and 1 × 10^−8^ M trichostatin A (TSA) for 48 h were taken using IN Cell analyzer 1000. Blue; Hoechst33342, Red; mCherry-MBD. (**D**, **E**) Concentration-dependent effects of 5-aza-dc (**D**) and TSA (**E**) on fluorescent MBD parameters and nuclei count of human iPSCs. Cells were treated with 10^−8^, 3 × 10^−8^, and 10^−7^ M 5-aza-dc, and 10^−9^, 3 × 10^−9^, and 10^−8^ M TSA for 48 h. Graphs show the ratios of each parameter ratio to the vehicle control. Data are expressed as the mean of the control ratio ± the standard error of three independent experiments. *P < 0.05; **P < 0.01 relative to the vehicle control.

A fusion gene consisting of the MBD region of MBD1, nuclear localization signal (nls), and the red fluorescent protein, mCherry, was introduced into human iPSCs (Fig. 1A). The mCherry-MBD-nls human iPSCs were used to establish a high-throughput screening system capable of evaluating the effects of chemicals on nuclear spatial distribution/concentration of DNA methylation (Fig. 1B). With the IN Cell Analyzer 1000, three fluorescent MBD parameters (MBD foci intensity, area, and count) were calculated. These values were used as indexes for the epigenetic effects of chemicals on human iPSCs (Fig. 1B). Hoechst 33342 positive nuclei number was also calculated for distinguishing MBD foci modulation activity with cytotoxicity of chemicals (Fig. 1B). The validity of this assay was confirmed using 5-aza-2′-deoxycytidine (5-aza-dc) and trichostatin A (TSA), which are known to modulate DNA methylation and histone acetylation in cells^27,28^. The mCherry-MBD-nls human iPSCs were treated with 5-aza-dc and TSA at three different concentrations for 48 h (Fig. 1C). We found that 5-aza-dc decreased MBD foci intensities, areas, and counts in a dose-dependent manner and TSA slightly decreased these parameters via HDAC inhibition, while nuclei count in 5-aza-dc and TSA exposure tendency decreased as possibly due to the cytotoxicity, but not significantly, indicating that 5-aza-dc and TSA reduced these parameters at the concentration without cytotoxicity. (Fig. 1D,E).

### Screening of 135 chemicals using mCherry-MBD-nls human iPSCs to modulate activities of DNA methylation

**Figure 2:**
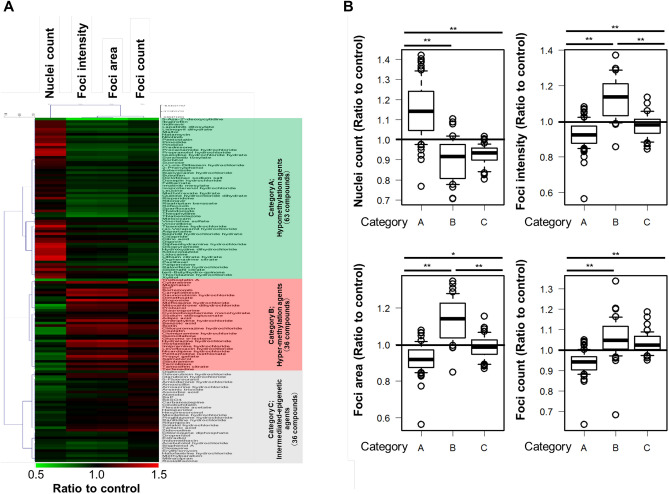
Screening of the modulating activities of 135 chemicals on DNA methylation using mCherry–MBD–nls human iPSCs. (**A**) Hierarchical cluster analysis of the effects of 135 chemicals on fluorescent MBD parameters. The 135 chemicals were divided into categories A (hypomethylation agents; green), B (hypermethylation agents; red), and C (intermediated epigenetic agents; gray), that decreased, increased, or had little or no effect on the fluorescent MBD parameters, respectively. (**B**) Box plots of the distributions of fluorescent MBD parameters in each category (*P < 0.05; **P < 0.01).

The human iPSCs-based screening system described in Fig. 1 was used to study the epigenetic toxicity against DNA methylation of 135 chemicals (Supplementary Table S1), including bisphenol A, benzo[*a*]pyrene (BaP), benzo[*c*]fluorene (BcF), beryllium sulfate (BeSO_4_), 5-aza-dc, TSA, and 129 chemicals, which is a focused collection of compounds with defined and diverse cardiotoxicity, such as arrhythmia, mitochondrial toxicity, fibrosis, and ion channel blockage, and contains various structurally and mechanistically different compound classes. The mCherry-MBD-nls human iPSCs were treated with several concentrations of 135 chemicals for 48 h. Hierarchical cluster analysis of the fluorescent MBD parameters (Supplementary Table S2) was performed and the 135 chemicals were categorized into three categories (Fig. 2A) as follows: category A (n = 63), reduced fluorescence intensities, areas, and counts of red fluorescent MBD foci and increased number of nuclei (Fig. 2B); category B (n = 36), increased fluorescence intensities, areas, and counts of red fluorescent MBD foci and decreased number of nuclei (Fig. 2B) and category C (n = 36), little or no effect on the fluorescent MBD parameters (Fig. 2B). Chemicals in category A were classed as hypomethylation agents, those in category B were classed as hypermethylation agents, and those in category C were classes as intermediated epigenetic agents.

### Machine learning-based physiological characterization using public databases

**Figure 3:**
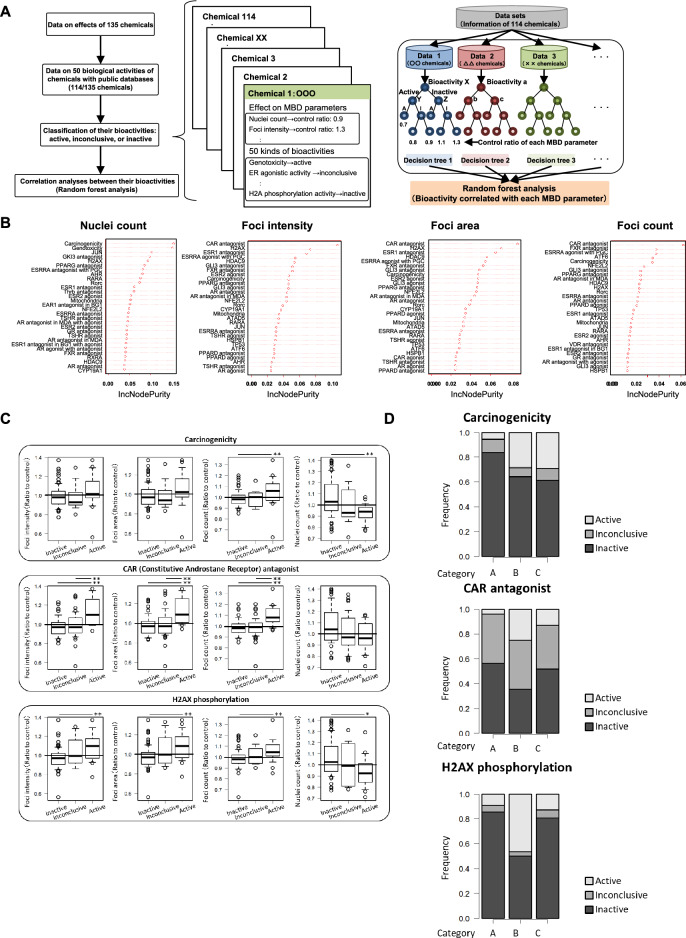
Machine learning-based physiological characterization using public databases. (**A**) Scheme of the random forest correlation analysis between epigenetic activities and physiological bioactivities of test compounds. The physiological bioactivity data were retrieved from the GENE-TOX, CCRIS, IARC monographs, and Tox21 databases. (**B**) Top 30 physiological bioactivities that highly correlated with the fluorescent MBD parameters according to the feature importance from the Increase in Node Purity. (**C**) Box plots of the distributions of fluorescent MBD parameters in chemicals with active, inconclusive, and inactive carcinogenicity, CAR antagonistic, and H2AX phosphorylation activities (**D**) The distributions of carcinogenicity, CAR antagonistic, and H2AX phosphorylation activities in categories A, B, and C. *P < 0.05; **P < 0.01.

We collected data on genotoxicity, carcinogenicity, and various physiological activities of 114 chemicals available from the GENE-TOX, Chemical Carcinogenesis Research Information System (CCRIS), International Agency for Research on Cancer (IARC) monographs and Toxicology in the 21st Century (Tox21) public databases (Fig. 3A, Supplementary Tables S3–S7) to better understand the functional difference among the hypomethylation agents, hypermethylation agents, and intermediated epigenetic agents. The GENE-TOX database contains data on various types of genotoxicity tests of chemicals. Data on carcinogenicity of numerous chemicals are available using CCRIS database and IARC monographs. The Tox21 database contains some types of agonistic or antagonistic activity against nuclear receptors or signaling pathways. The activity of chemicals was categorized for active, inactive, or inconclusive (activity that cannot be determined if there is activity or not) (Supplementary Tables S3–S7). We then analyzed the correlations between four fluorescent MBD parameters and 50 physiological activities using a random forest algorithm. Figure 3B shows the top 30 bioactivities were highly correlated with each parameter, ranked according to the feature importance from Increase in Node Purity. Carcinogenicity and genotoxicity were highly correlated with nuclei count, while the activities of CAR antagonists, H2AX phosphorylating chemicals, and estrogen receptor 1 antagonists were highly correlated with MBD foci intensity, area, and count. Quantitative analysis showed that the activities of CAR antagonists and H2AX phosphorylating chemicals increased the intensities, areas, and counts of fluorescent MBD foci compared with inactive or inconclusive chemicals (Fig. 3C). Figure 3D shows the proportion of inactive, inconclusive, or active chemicals with CAR antagonistic or H2AX phosphorylation activities among hypomethylation agents, hypermethylation agents, and intermediated epigenetic agents. The highest percentages of active CAR antagonists and H2AX phosphorylating agents were observed in hypermethylation agents. The CAR antagonistic and H2AX phosphorylation activities of eight chemicals with the highest activities of fluorescent MBD foci parameters in hypomethylation and hypermethylation agents, respectively, and bisphenol A in intermediated epigenetic agents are presented in Table 1. Hypermethylation agents including etoposide, daunorubicin, and cytarabine were active for both CAR antagonistic and H2AX phosphorylation activities, whereas most hypomethylation agents or intermediated epigenetic agents were inactive. These data indicate a functional connectivity between the fluorescence of MBD foci parameters and physiological activities, such as CAR antagonistic and H2AX phosphorylation activities, of human iPSCs.

**Table 1:** Effects of hypomethylation, hypermethylation, and intermediated epigenetic agents on fluorescent MBD foci parameters and physiological activities.

Category	Chemical	Major use	Ratio to control			Activity	
			Foci intensity	Foci area	Foci count	CAR antagonist	H2AX phosphorylation
A; Hypomethylation agents	5-Aza-2′-deoxycytidine	Drug	0.56	0.56	0.63	Inconclusive	Inactive
	Thiabendazole	Drug	0.77	0.77	0.85	Inconclusive	Inactive
	Theophylline	Natural compound	0.80	0.83	0.89	Inactive	Inactive
	Procainamide hydrochloride	Drug	0.84	0.83	0.85	Inactive	Inactive
	Thalidomide	Drug	0.85	0.85	0.95	Inactive	Inactive
	Lisinopril dihydrate	Drug	0.85	0.84	0.88	Inactive	Inactive
	Nilotinib	Drug	0.86	0.85	0.87	–	–
B; Hypermethylation agents	Etoposide	Drug	1.37	1.35	1.34	Active	Active
	Hydralazine hydrochloride	Drug	1.30	1.33	1.20	Inconclusive	Inconclusive
	Propyl gallate	Food additives	1.29	1.31	1.15	Inconclusive	Active
	Bortezomib	Drug	1.29	1.27	1.10	Active	Inactive
	Daunorubicin hydrochloride	Drug	1.29	1.27	1.10	Active	Active
	Amitriptyline hydrochloride	Drug	1.28	1.29	1.11	Inconclusive	Inactive
	Cytarabine	Drug	1.26	1.25	1.06	Active	Active
	Biotin	Nutrient	1.23	1.23	1.16	Inactive	Inactive
C; Intermediated-epigenetic agents	Bisphenol A	Industrial material	0.89	0.89	0.95	Inconclusive	Inactive

– Data were not existing.

### Effects of hypermethylation agents on genome-wide and gene-specific DNA methylation patterns in human iPSCs

**Figure 4:**
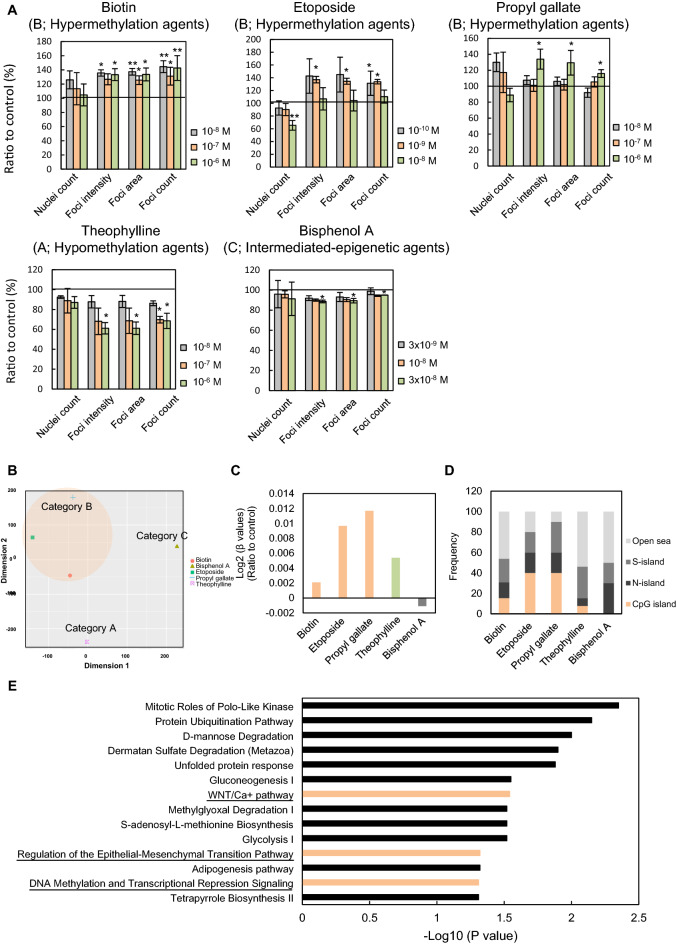
Effect of hypermethylation agents on genome-wide and gene-specific DNA methylation patterns in human iPSCs. (**A**) Concentration-dependent effects on fluorescent MBD parameters of human iPSCs. Cells were treated with 1 × 10^−8^, 1 × 10^−7^, and 1 × 10^−6^ M biotin, 1 × 10^−10^, 1 × 10^−9^, and 1 × 10^−8^ M etoposide, and 1 × 10^−8^, 1 × 10^−7^, and 1 × 10^−6^ M propyl gallate (category B, hypermethylation agents); 1 × 10^−8^, 1 × 10^−7^, and 1 × 10^−6^ M theophylline (category A, hypomethylation agent); and 3 × 10^−9^, 1 × 10^−8^, and 3 × 10^−8^ M bisphenol A (category C, intermediated epigenetic agent) for 48 h. Graphs showed the ratios of each fluorescent MBD parameter ratio to the vehicle control. Data were expressed as the means of the control ratio ± the standard error of three independent experiments. *P < 0.05; **P < 0.01 relative to the vehicle control. (**B**) t-SNE analysis of β-values relative to the vehicle control. (**C**) Changes in DNA methylation rates at all sites. Data are presented as the mean of log2 (β-values) ratio to the vehicle control. (**D**) Distributions of the genome area of differential methylated site relative to the vehicle control. (**E**) Top canonical pathways associated with the common hypermethylated genes with a fold change threshold of more than 10 following treatment with etoposide or propyl gallate using Ingenuity Pathway Analysis. − Log10 (*P* values) of statistically significant pathways (*P* < 0.05) are presented. See also Table S9.

The effects of the chemicals on DNA methylation were evaluated using a DNA methylation array. Five compounds were selected for the array. Etoposide and propyl gallate, which are active or inconclusive against CAR antagonistic and H2AX phosphorylation activities, and biotin, which is a B vitamin with significant effects on DNA hypermethylation, were selected as representative hypermethylation agents. Theophylline, a bitter ingredient in tea leaves, and bisphenol A, suspected to induce DNA hypomethylation^29^, were selected as representative hypomethylation agents and intermediated epigenetic agents, respectively. The mCherry-MBD-nls human iPSCs were treated with biotin, etoposide, propyl gallate, theophylline, or bisphenol A at three different concentrations for 48 h. Consistent with the results of the screening analysis, hypermethylation agents increased the values of the three parameters of fluorescent MBD foci, while theophylline and bisphenol A decreased or showed limited effects on these parameters (Fig. 4A and Supplementary Fig. S1).

We next examined the effects of these chemicals on genome-wide DNA methylation rates in mCherry–MBD–nls human iPSCs using Infinium MethylationEPIC BeadChip Kit. T-distributed stochastic neighbor embedding (t-SNE) plots indicated that the methylation profiles of three hypermethylation agents tend to show similar patterns, because the plots of three hypermethylation agents are located in relatively close proximity compared to hypomethylating agents and intermediate epigenetic agents (Fig. 4B). Further, all three hypermethylation agents (biotin, etoposide, and propyl gallate) were found to increase the average DNA methylation level. But, unfortunately the hypomethylating agent (theophylline) did not decrease the average DNA methylation level (Fig. 4C). The distribution of the genome area of differentially methylated sites revealed that the proportions of CpG islands regulated by hypermethylation agents (especially etoposide and propyl gallate) were higher than those regulated by hypomethylation agents and intermediated epigenetic agents (Fig. 4D, Supplementary Table S8). To explore the biological interpretation of the epigenetic effects of the hypermethylation agents, etoposide and propyl gallate, pathway analysis was performed with the common hypermethylated genes with a fold change threshold of more than 10 following treatment with etoposide or propyl gallate using knowledge-based Ingenuity Pathway Analysis (Supplementary Table S9). Canonical pathway analysis revealed the enrichment for genes regulating epigenetic gene regulation pathway, such as “DNA methylation and Transcriptional Repression Signaling”, and stemness-related pathway, such as “Wnt/Ca + pathway” and “Regulation of the Epithelial-Mesenchymal Transition pathway” (Fig. 4E). These results suggested a potential effect of the hypermethylation agents with CAR antagonistic and H2AX phosphorylation activities such as etoposide and propyl gallate on stemness-related signaling pathways by regulating the DNA hypermethylation at the CpG regions.

### Effects of hypermethylation agents on the levels of gene expression and DNA methylation of stemness-related genes in human iPSCs

**Figure 5:**
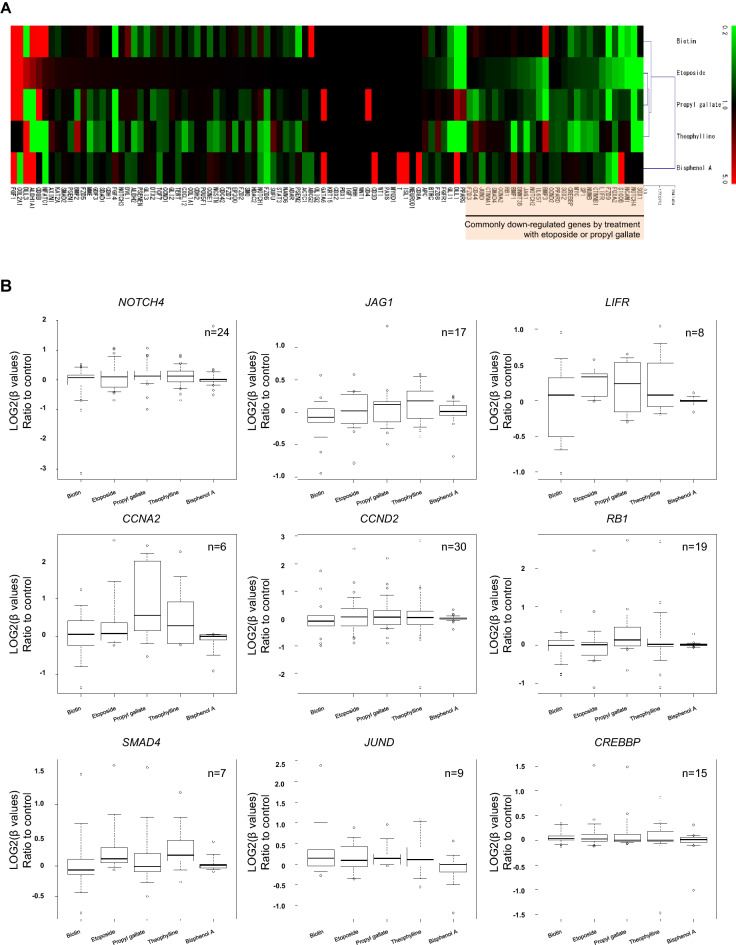
Effects of hypermethylation agents on the levels of gene expression and DNA methylation of stemness-related genes in human iPSCs. (**A**) Hierarchical cluster analysis of the gene expression profiles of 100 stemness-related genes in human iPSCs. Cells were treated with biotin, etoposide, and propyl gallate (category B, hypermethylation agents); theophylline (category A, hypomethylation agent); and bisphenol A (category C, intermediated epigenetic agent) for 48 h. Data are presented as the fold change of gene expression relative to the vehicle control. (**B**) Effects of hypermethylation agents on DNA methylation rate at CpG sites around the locus of *NOTCH4*, *JAG1*, *LIFR*, *CCNA2*, *CCND2*, *RB1*, *SMAD4*, *JUND*, and *CREBBP* genes. Data are presented as log2 (β-values) ratio to the vehicle control. The total number (n) indicates the number of CpG sites in each gene. See also Table S11.

Then, we evaluated the effects of chemicals on the expression of 100 stemness-related genes using TruSeq Targeted RNA Stem Cell Panel Kit. Hierarchical cluster analysis on the gene expression profiles (Supplementary Table S10) showed that etoposide and propyl gallate had similar effects on gene expression compared with biotin and chemicals in the other categories (Fig. 5A), similar to their effects on DNA methylation.

Notably, the expression of 28 out of 100 genes were commonly decreased by treatment with etoposide and propyl gallate (Fig. 5A). We evaluated the DNA methylation rate at each CpG site around the locus of these 28 genes to examine whether the downregulation of these genes was associated with gene locus-specific DNA methylation. Among the 28 genes, the mean DNA methylation rates at the CpG sites of 16 genes were increased by either etoposide or propyl gallate. Especially, the mean DNA methylation rates at the CpG sites of nine genes, *NOTCH4*, *JAG1*, *LIFR*, *CCNA2*, *CCND2*, *RB1*, *SMAD4*, *JUND*, and *CREBBP*, were increased after treatment with etoposide and propyl gallate (Fig. 5B, Supplementary Table S11).

### Validation of effects of chemicals on DNA methylations by RRBS

**Figure 6:**
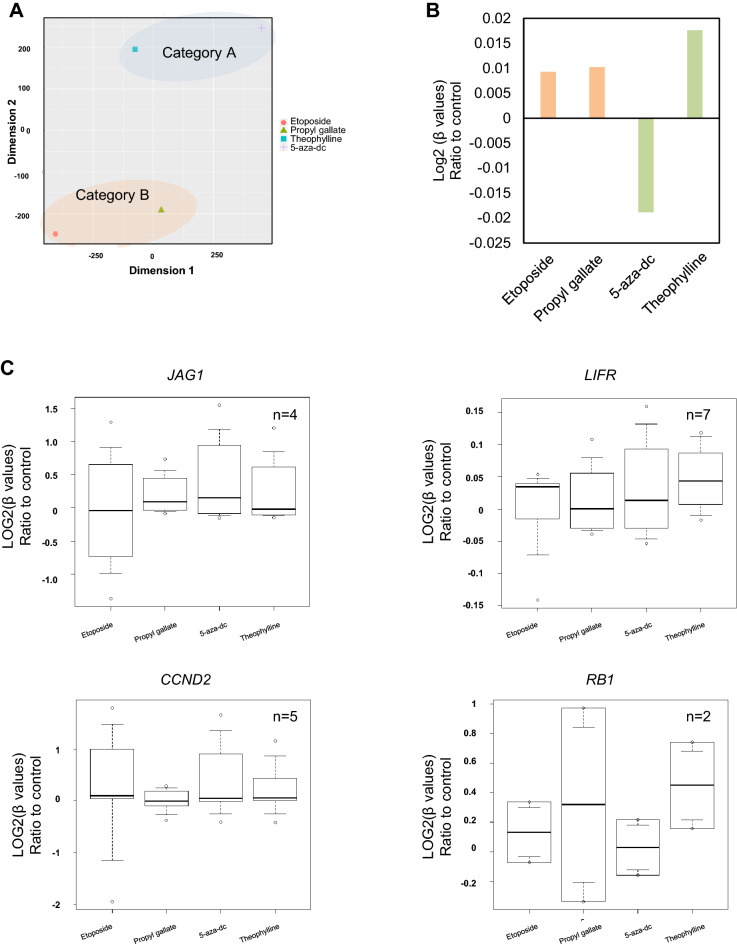
Validation of effects of chemicals on DNA methylations by RRBS. (**A**) t-SNE analysis of β-values relative to the vehicle control. (**B**) Changes in DNA methylation rates at all sites. Data are presented as the mean of log2 (β-values) ratio to the vehicle control. (**C**) Effects of hypermethylation agents on DNA methylation rate at CpG sites around the locus of *JAG1*, *LIFR*, *CCND2*, and *RB1* genes. Data are presented as log2 (β-values) ratio to the vehicle control. The total number (n) indicates the number of CpG sites in each gene. See also Table S12.

Finally, we validated the effects of chemicals on DNA methylation by performing Reduced Representation Bisulfite Sequencing (RRBS), one of bisulfite sequencings that can measure DNA methylation levels in the regions of 3–5 million CpG islands. We evaluated the effects of 4 chemicals, etoposide and propyl gallates as hypermethylation agents and 5-aza-dc and theophylline as hypomethylation agents. T-SNE plots indicated that the methylation profiles of hypermethylation agents and hypomethylation agents tend to show similar patterns each other (Fig. 6A). Further, both hypermethylation agents (etoposide, and propyl gallate) were found to increase the average DNA methylation level in agreement with the data in DNA methylation array (Fig. 6B). Regarding hypomethylation agents, we observed that 5-aza-dc significantly reduced the average DNA methylation level and its fluorescent MBD-modulating activity. In contrast, theophylline did not decrease the average DNA methylation levels, consistent with the results from the EPIC array. This suggests that theophylline may act through a MBD-independent epigenetic mechanism (Fig. 6B). Then, we evaluated the mean DNA methylation rates at the CpG sites of 9 genes that expression levels and DNA methylation levels at the CpG sites in DNA methylation array were decreased and increased respectively by either etoposide or propyl gallate (Fig. 5B). Among 9 genes, the mean DNA methylation rates at the CpG sites of four genes, *JAG1*, *LIFR*, *CCND2*, and *RB1*, were increased after treatment with etoposide and propyl gallate like DNA methylation array (Fig. 6C, Supplementary Table S12), indicating that these changes in gene expression were correlated with their DNA methylation status via a MBD-dependent mechanism.

## Discussion

**Figure 7:**
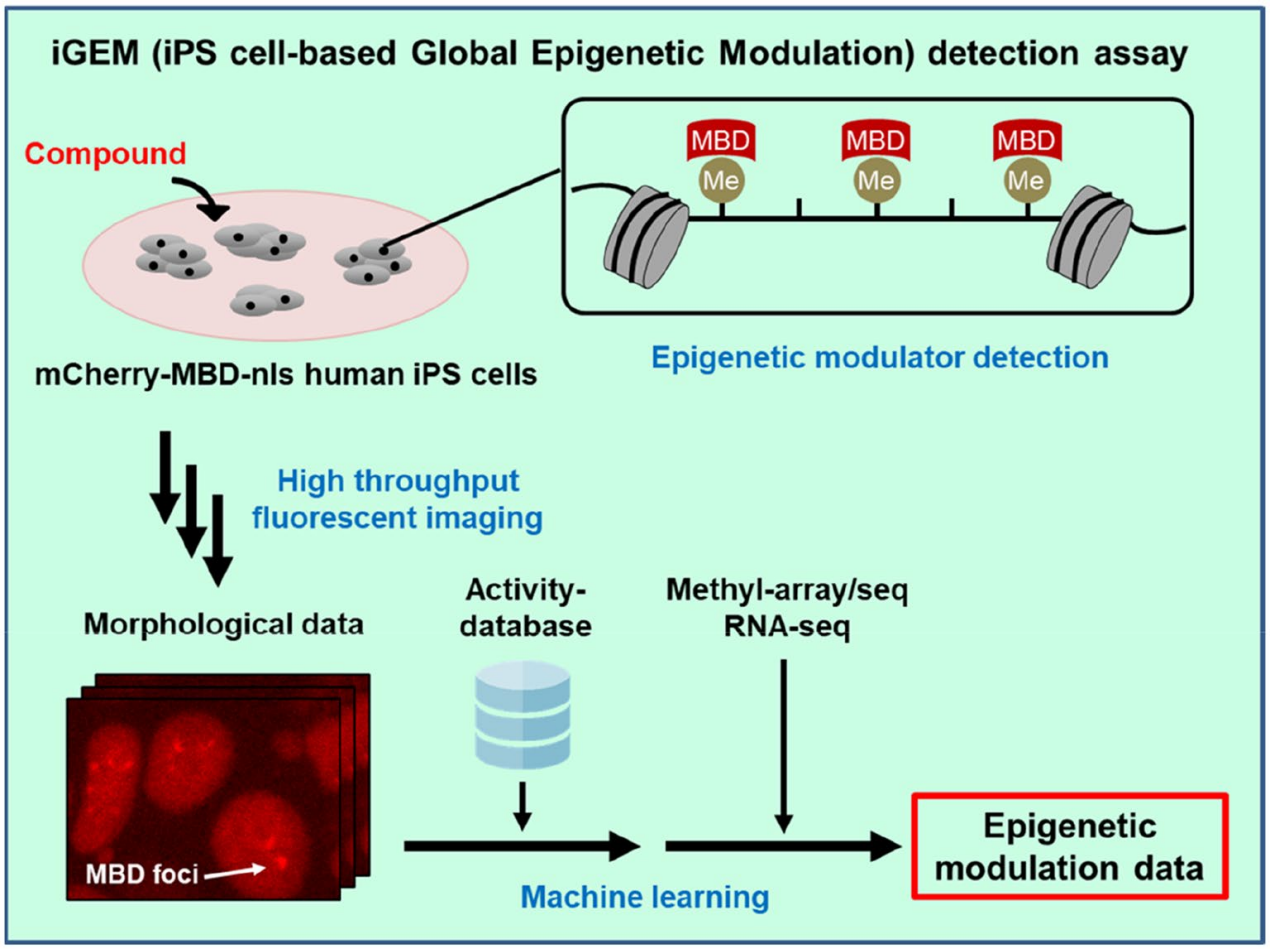
Schematic overview of iPS cell-based global epigenetic modulation (iGEM) detection assay.

In the present study we established an iGEM assay, which is a high-throughput system to detect epigenetic teratogens/mutagens using human iPSCs harboring a methyl-CpG-binding domain (MBD) fused to a fluorescent protein (Fig. 7). Most of existing assays to evaluate epigenetic effects of chemicals were enzyme-based assays^14–16^ and the reaction between chemicals and enzymes does not always match the reaction in intracellular events or in vivo. In contrast, the iGEM assay using human iPSCs has some advantages. For instance, it can: (1) mimic actual human effects, (2) be stably used for a long period of time because human iPSCs proliferate infinitely, and (3) detect epigenetic effects very sensitively because open chromatin regions are more frequent in iPSCs compared to somatic cells. Our study is the first time that the application of fluorescence MBD proteins as an indicator to quantitatively evaluate the epigenetic effect of chemicals on the DNA methylation of human iPSCs within a high-throughput screening system. Additionally, the iGEM assay can classify chemicals according to the MBD signal intensity, which reflects their degree of nuclear spatial distribution/concentration of DNA methylation. Our MBD-based screening system is thus a powerful assay to detect epigenetic toxicants within different contexts, including environmental monitoring and pharmaceutical development.

A change in fluorescence signal can be caused by changes in chromatin compaction in the drug treated cells. Our previous study demonstrated that the fluorescent MBD construct could be used to monitor DNA methylation in situ and the formation and rearrangement of methylated heterochromatin under physiological conditions^20^. On the other hands, it was reported that MBD can bind to methylated cytosine not only in the heterochromatin region, but also in the promoter/enhancer/gene center region^19^. In this study, most of the MBD spots co-localized with Hoechst 33342 spots, but not all of them did. As a result of technical limitations in our current pipeline, it was relatively difficult to detect and distinguish each Hoechst 33342 spot. Further studies using more sensitive and sophisticated systems may provide additional insights into the relationships between MBD spots and Hoechst 33342 spots. Due to the fact that the mCherry-MBD-nls hiPSCs cells used in this study express MBD under the control of the CAG promoter and maintain a highly expressed state, indicating that the effects of chemical exposure on MBD expression and protein stability are likely low. However, we cannot completely rule out the possibility that chemical exposure may affect the stability or expression level of the MBD protein, leading to changes in fluorescence intensity. Therefore, we carefully selected the concentration of the target chemicals to avoid high cytotoxicity and evaluated the effects of chemicals on global DNA methylation using various MBD indicators, as well as EPIC array and RRBS analyses. In fact, with chemicals classified as category B hypermethylation agents, we observed an increase in MBD foci intensity, foci area, and foci count, as well as an increase in global DNA methylation by EPIC array and RRBS analyses. Therefore, it is reasonable to investigate the effects of chemicals on global DNA methylation using various MBD indicators.

The global epigenetic toxicity of 135 chemicals, most of which are suspected to have cardiotoxicity were evaluated in this study^30–33^. We identified 36 chemicals that caused hypermethylation, and 63 chemicals that caused hypomethylation, as evidenced by their effects on MBD foci intensity, area, and count. These findings provide clear evidence that chemicals with cardiotoxicity can directly affect DNA methylation in human iPSCs, highlighting the importance of comprehensively examining the epigenetic effects of drug exposure during early life stages. The stemness stage, during which the pluripotency of iPSCs or ESCs is regulated by DNA methylation, is particularly sensitive to external factors, making it crucial to study the epigenetic effects of chemical exposure. This feature of iPSCs and ESCs is convenient for detecting the epigenetic activity of chemicals, as we previously reported^25^. Random forest-based correlation analysis using public databases provided mechanistic insight and highlighted CAR antagonist and H2AX phosphorylation activities as the two physiological activities mostly correlated with fluorescent MBD parameters. H2AX phosphorylation is a well-known marker for DNA damage, and a relationship between H2AX phosphorylation and epigenetic toxicity has been reported^34^, thus further demonstrating that our evaluation system can be used to evaluate the epigenetic toxicity of chemicals.

Much attention has been focused on chemicals disturbing the state of global DNA methylation^14–16,35^. In the present study, we showed that biotin, etoposide, and propyl gallate increased the overall DNA methylation rate, which was consistent with their effects on fluorescent MBD parameters using mCherry-MBD-nls engineered human iPSCs. Previous studies have shown that etoposide inhibits topoisomerase, which regulates DNA methylation^36,37^. The target proteins of etoposide, TOP2A and TOP2B, bind to histone deacetylases HDAC1 and HDAC2^38^, which have been shown to form a heterochromatic complex with MBD1^39^. Although propyl gallate is widely used as antioxidant additives in many cosmetic products^40^, high concentrations of propyl gallate is considered to be toxic to the liver^41^. Our data showed for the first time that its epigenetic toxicity occurs via MBD1-mediated transcriptional repression. Biotin also induced DNA hypermethylation in the present study. Biotin is an essential water-soluble vitamin that is highly involved in the maintenance of normal cell function and biotinylation of lysine resides in histones has been shown to be a chemical modification of histones and may be involved in diet-related epigenetic regulation^42,43^.

Of the two hypomethylation agents (5-aza-dc and theophylline), only 5-aza-dc consistently reduced both fluorescent MBD activity and DNA methylation levels, while theophylline increased DNA methylation but not fluorescent MBD activity. Theophylline has been shown to have histone deacetylase activity, which may contribute to its MBD-independent hypermethylation effect^44^. However, there have been no reports of a direct relationship between theophylline and MBD proteins, and further studies are needed to understand the mechanisms of action of theophylline. Additionally, more studies using a larger number of agents influencing stemness or interacting stemness factors^45–49^ are needed to determine the usefulness of our system for detecting hypomethylation agents.

In conclusion, the present study presents an integrated analytical strategy combined with chemical screening, machine learning-based physiological characterization, genome-wide DNA methylation and gene expression profiling, and knowledge-based pathway analysis using mCherry-MBD-nls engineered human iPSCs. With this analytical strategy, we identified and functionally characterized the impact of hypermethylation agents with CAR antagonistic and H2AX phosphorylation activities, such as etoposide and propyl gallate on stemness-related signaling pathway by regulating the DNA hypermethylation at the CpG regions. With more data on other types of chemicals, our DNA methylation assay may have the potential for use in not only toxicological evaluation but also drug discovery to identify DNA methylation-modulating chemicals. The data generated in the present study may serve as a resource and framework for examining the quantitative influence of chemicals against DNA methylation and exploring their underlying MBD1-dependent molecular mechanisms. While our study provides a novel screening method for chemicals that affect DNA methylation, there are several limitations to our study. First, we used a limited number of chemicals and did not examine all possible categories of chemicals that could affect DNA methylation. Second, our study used a cell line model and did not examine the effects of these chemicals on DNA methylation in other cell types or in vivo. Third, while we used various MBD indicators to investigate the effects of chemicals on DNA methylation, we did not directly measure the binding affinity of MBD to methylated DNA. Further studies are required to expand the application and validate the performance of this high-throughput epigenetic evaluation system in pharmaceutical development and risk assessment of public health.

## Methods

### Chemicals

Dimethyl sulfoxide (DMSO) was purchased from Sigma-Aldrich (St. Louis, MO, USA), and 5-aza-2′-deoxycytidine (5-aza-dc), trichostatin A (TSA), bisphenol A, benzo[*a*]pyrene (BaP), benzo[*c*]fluorene (BcF), and beryllium sulfate (BeSO_4_) were obtained from Wako Pure Chemical Industries, Ltd. (Osaka, Japan). The SCREEN-WELL Cardiotoxicity Library was purchased from Enzo LifeScience Inc. (Supplementary Table S1). All chemicals were dissolved in DMSO and diluted in culture media. The final concentration of DMSO did not exceed 0.1%.

### Culture of human iPSCs

The human iPSC 201B7 cell line^50^ was provided by Riken BRC Cell Bank (Cell No. HPS0063) and seeded on SNL 76/7 feeder cells (European Collection of Authenticated Cell Cultures ECACC, Salisbury, UK) and maintained using Primate ES Cell Medium (ReproCELL Inc., Kanagawa, Japan), which is an undifferentiated maintenance culture medium for primates. When the colonies reached a size of ≥ 500 µm in diameter, the cells were separated in the form of colonies with a human ES cell exfoliation solution (ReproCELL) and preserved with the cell preservation solution Cell Reservoir One (Nacalai), which is a vitrification method. Epigenetic assays were performed after thawing the cells to ensure the iPSCs were in a feederless state. The cells were then seeded on a dish coated with extracellular basement membrane substrate and maintained in the undifferentiated maintenance culture medium mTeSR-1 medium (STEMCELL Technologies, Vancouver, Canada) in a dish coated with Matrigel (BD Biosciences, San Jose, CA) and cultured at 37 °C with 5% CO_2_. The medium was changed every day, and the cells were passaged every week using ReLeSR (STEMCELL Technologies). The cells were grown to a density of 80–90% and then treated with ReLeSR (StemCellTechnologies), which is a colony stripping solution, to obtain an appropriate colony size, and then subcultured and maintained. The cells were maintained for three generations and then used in experiments.

### Isolation of human iPSCs carrying the mCherry-MBD-nls construct

The mCherry-MBD-nls construct (Supplementary Fig. S1) was introduced to human iPSCs using modified reverse transfection method with Lipofectamine 2000 (Thermo Fisher Scientific, Tokyo, Japan). Briefly, 1 µg of pPB-CAG-mCherry-MBD-nls and 2 μg of hyPBase vector (pCAG-hyPBase)^51^ were mixed in 100 µL of Opti-MEM (Thermo Fisher Scientific), and 3 μL of Lipofectamine 2000 was then added to the Opti-MEM solution to prepare the DNA–lipid complex. The iPSCs cultured under feeder-free conditions were dissociated into single cells using Accutase (Sigma-Aldrich, St. Louis, MO) and the cell pellet was obtained by centrifugation. The DNA–lipid mixture was then added to the cell pellet and mixed gently. The cell suspension was incubated at room temperature for 20 min, and the DNA–lipid-cell suspension was plated into dishes containing the culture medium with 10 μM of Y-27632 (Wako Pure Chemical), and incubated at 37 °C. After a 4-h incubation, the medium was replaced with fresh medium containing 10 μM of Y-27632 and the cells were further incubated overnight. The next day, the medium was replaced with fresh medium without Y-27632. Two days after transfection, 10 μg/mL blasticidin (Kaken Pharmaceutical Co., Ltd., Tokyo, Japan) was added to the medium. After a drug selection period of 5–7 days, mCherry-positive colonies were picked and genotyped to confirm the presence of the reporter construct.

### Chemical screening for MBD fluorescent modulating activity

mCherry-MBD-nls human iPSCs were seeded on a Laminin511-E8 (iMatrix-511, Nippi, Tokyo, Japan)-coated 96-well plate at a density of 7.2 × 10^3^ cells/well on day 0, and the medium was changed on day 1. On day 2, cells were exposed to each chemical substance at the preliminarily validated concentrations (1 × 10^−6^, 1 × 10^−7^, 1 × 10^−8^, 3 × 10^−8^, 1 × 10^−9^, and 1 × 10^−10^ M), at which the cell viability was more than 70% compared with control well, which was treated with 0.1% DMSO for 48 h. Specifically, bortezomib, camptothecin, and vincristine sulfate were added at a concentration of 1 × 10^−10^ M, amsacrine hydrochloride, daunorubicin hydrochloride, doxorubicin hydrochloride, etoposide, idarubicin hydrochloride, isoproterenol hydrochloride, mitoxantrone dihydrochlorides, paclitaxel, and staurosporine were added at a concentration of 1 × 10^−9^ M, bisphenol A was added at a concentration of 3 × 10^−8^ M, cinobufotalin, cytarabine, digitoxin, digoxin, methotrexate hydrate, TSA, and vinorelbine were added at a concentration of 1 × 10^−8^ M, 5-aza-dc was added at concentration of 1 × 10^−7^ M, and the other 114 chemicals were added at a concentration of 1 × 10^−6^ M (Supplementary Table S2). After exposure to the drugs (day 4), cells were fixed using 4% paraformaldehyde for 15 min and nuclei were stained with Hoechst33342 (Life Technologies, Carlsbad, CA, USA) for 15 min at room temperature. Cells were washed three times with phosphate-buffered saline and stored at 4 °C in the dark until analyzed.

### Image acquisition and analysis of fluorescent signals

Image acquisition and analysis were performed using IN Cell Analyzer 1000 (the current name Cytiva Tokyo, Japan was previously known as GE Healthcare UK Ltd.) as previously described^21^. Briefly, immunofluorescent images (nine fields per each well in a 96-well plate) were obtained using an automated IN Cell Analyzer 1000 (20× objective lens). Excitations and emissions were recorded for the blue and red channels to detect the Hoechst 33342 (nuclei count) and three fluorescent MBD parameters (foci intensity, area, and count), respectively. Obtained immunofluorescent images were analyzed using image analysis algorithms generated using the IN Cell Developer Tool Box software (v1.7) in four steps: “cell segmentation”, “nuclear segmentation”, “foci segmentation”, and “measure nodes”. Specifically, each cell was distinguished from the other cells and the background using a cell segmentation algorithm, followed by nuclear segmentation and foci segmentation processes to distinguish the individual MBD parameters, nucleus, and cells. During segmentation of the targeted parameters, the measure node process was performed to quantify each target parameter as “nuclei count”, “foci intensity/nucleus”, “foci area/nucleus” and “foci count/nucleus”. Three fluorescent MBD parameters (foci intensity, area, and count) were values per nucleus.

The effects of each chemical on the fluorescent MBD parameters (nuclei count and foci intensity, area, and count) were expressed as a ratio to the values of a control treated with 0.1% DMSO. Based on these values, hierarchical cluster analysis was performed using Multiple Experiment Viewer (MeV) software^52^, which is a freely available java tool for genomic data analysis (ver. 4.9) (http://mev.tm4.org/). Pearson correlation and average linkage clustering were selected for the distance metric and linkage method in this analysis, respectively.

### Correlation between physiological and fluorescent MBD-modulating activities of chemicals

Data on genotoxicity, carcinogenicity, and various physiological activities reported for each chemical were collected from the GENE-TOX, CCRIS, IARC monographs, and Toxicology in the 21st Century (Tox21) public databases^53^. GENE-TOX is a dataset of genetic toxicology test data from peer-reviewed and open access scientific literature for > 3000 chemicals that was developed by the United States Environmental Protection Agency. Data on genotoxicity of tested chemicals from GENE-TOX are available at https://www.nlm.nih.gov/databases/download/genetox.html. The GENE-TOX database contained data on various types of experiment to evaluate genotoxicity of chemicals, such as bacterial assay, mammalian cell mutation assay, chromosomal mutation assay, and in vitro and in vivo indicator tests. CCRIS was developed by the National Cancer Institute and is a collection of test results and conditions for carcinogenicity, mutagenicity, tumor promotion, and tumor inhibition of more than 8,000 chemicals, with using animal (such, as rat or mouse) studies. Data on toxicity of each chemical from CCRIS are available at https://www.nlm.nih.gov/databases/download/ccris.html. IARC is a specialized agency of the World Health Organization that provides IARC monographs on the identification of carcinogenic hazards to humans at https://monographs.iarc.who.int/agents-classified-by-the-iarc/. Tox21 program is a unique collaboration between several federal agencies, such as the National Institute of Environmental Health Sciences, National Toxicology Program, National Center for Advancing Translational Sciences, U.S. Food and Drug Administration, and National Center for Computational Toxicology, and evaluates some types of physiological effects of a diverse range of chemical substances. Data on each physiological activity of 135 chemicals used in this study were collected from PubChem (https://pubchem.ncbi.nlm.nih.gov/). The Tox21 database contains various types of data of tested chemicals, agonistic or antagonistic activity against nuclear receptors, such as estrogen receptor, androgen receptor, or peroxisome proliferator activated receptor, and modulating activity of some signaling pathways, such as sonic hedgehog signaling pathway, p53 signaling pathway, or retinoid-related orphan receptor gamma signaling pathway. The activity of chemicals was categorized for active, inconclusive, or inactive according to the criteria described below (Supplementary Tables S3–S7). For genotoxicity, chemicals positive for at least one experiment in the GENE-TOX database were categorized as active, whereas those negative for all experiments were categorized as inactive. Regarding carcinogenicity, activity was determined by combing the results from the IARC and CCRIS database. In the IARC database, chemicals belonging to Groups 1 or 2A were categorized as active. Among the IARC chemicals in Groups 2B or 3, which were positive in at least one animal experiment in CCRIS database or negative for all animal experiments, were categorized for active or inconclusive, respectively. Chemicals in Group 4 and those lacking IARC data were categorized as active or inactive when they showed positive data in at least one animal experiment or negative data in all animal experiments in CCRIS, respectively. For other physiological activities, data were obtained from the Tox21 database using the PubChem website. After obtaining data for the original compounds and its hydrochlorides, the activity was determined by considering these two data as follows: when one was active and the other was inconclusive or inactive, activity was categorized for active or inconclusive, respectively, and, when one was inactive and the other was inconclusive, the activity was categorized as inactive. These category data were combined with data for four fluorescent MBD parameters of each chemical. The randomForest R package was used to rank each physiological activity correlated with fluorescent MBD parameters^54^.

### DNA methylation array

Total genomic DNA was extracted from human iPSCs treated with 0.1% DMSO (control), 1 µM biotin, 1 nM etoposide, 1 µM propyl gallate, 1 µM theophylline, or 30 nM bisphenol A for 48 h using DNeasy Blood & Tissue Kit (QIAGEN, Hilden, Germany) according to the manufacturer’s protocol. DNA methylation array analysis was assessed using Infinium MethylationEPIC BeadChip Kit (Illumina, Inc., CA, USA) and conducted at Riken Genesis Co., LTD. (Kawasaki, Japan)^55^. Briefly, bisulfite conversion of isolated genomic DNA was performed using EZ-DNA Methylation Kit (ZYMO RESEARCH, CA, USA) and the genomic DNA samples were amplified, fragmented, and hybridized to Illumina Infinium Human MethylationEPIC Beadchip using standard Illumina protocol. Samples were reacted with labeled nucleotide and then scanning of the BeadChip was performed using the Illumina iScan system (Illumina). The obtained scan data and genome studio software (V2011.1, Methylation Module 1.9.0, Illumina) were used to detect genes (detection *P*-value < 0.05) suitable for further analysis. The DNA methylation rate of each detected methylation site was scored as β-values using genome studio software. Differentially hypermethylated sites were defined as the sites where the value of DiffScore was > 13 (*P* < 0.05) (Supplementary Table S8). t-SNE plots of fold changes of each β-value compared with the control in the chemical treatment samples were created using iDEP.96 software (http://bioinformatics.sdstate.edu/idep/)^56^. Pathway analysis was conducted with QIAGEN Ingenuity Pathway Analysis (QIAGEN) (Supplementary Table S9). Distributions of the genome area of differential methylated site of each chemical and DNA methylation rate at CpG sites around the locus of *NOTCH4*, *JAG1*, *LIFR*, *CCNA2*, *CCND2*, *RB1*, *SMAD4*, *JUND*, and *CREBBP* genes were created according to classification of UCSC RefGene Group using genome studio software (Supplementary Table S11). And graphs were created using EZR (Saitama Medical Center, Jichi Medical University; http://www.jichi.ac.jp/saitama-sct/SaitamaHP.files/statmedEN.html)^57^, which is a graphical user interface for R (The R Foundation for Statistical Computing, Vienna, Austria, version 3.5.2). Specifically, it is a modified version of R commander (version 1.38) designed to add statistical functions frequently used in biostatistics.

### RNA sequencing

RNeasy Micro Kit (QIAGEN) was used to isolate total RNA from human iPSCs treated with 0.1% DMSO (control), 1 µM biotin, 1 nM etoposide, 1 µM propyl gallate, 1 µM theophylline, or 30 nM bisphenol A for 48 h according to manufacturer’s instructions. A library of each RNA sample was prepared using TruSeq Targeted RNA Expression Kit (Stem Cell Panel, Illumina) according to the manufacturer’s protocol. RNA sequencing analysis using Miseq instrument (Illumina) was conducted using the library with Miseq Reagent Micro Kit v2 (300 Cycles, Illumina). MiSeq Control Software was used for basecalling^58^. Next, the annotation (Human hg19, UCSC) and quantification and normalization (reads per kilobase of exon per megabase of library size, RPKM) of each FASTQ data was performed for further data analysis. Hierarchical cluster analysis according to fold change in RPKM values of genes compared with control sample was performed using MeV software (Supplementary Table S10). Euclidean distance and average linkage clustering were selected for distance metric and linkage methods in this analysis, respectively.

### Reduced Representation Bisulfite Sequencing (RRBS)

Total genomic DNA was extracted from cells treated with 0.1% DMSO (control), 1 nM etoposide, 1 µM propyl gallate, 1 µM theophylline, or 10 nM 5-aza-dc through Blood & Cell Culture DNA Mini Kit (QIAGEN) according to the manufacturer’s protocol. RRBS was conducted at Rhelixa Co., Ltd. (Tokyo, Japan).

Briefly, after quality check of DNA samples, a library of each sample was prepared using Zymo Research Zymo-Seq RRBS Library Kit (ZYMO RESEARCH, D5461, Ver. 1.0.0) according to the manufacturer’s protocol. Then, sequences of the library samples (FASTQ files) were obtained using a Illumina NovaSeq 6000 (Illumina).The quality of the obtained raw paired-end sequence reads (150 bp × 2 paired-end) was evaluated using FastQC (Version 0.11.7; https://www.bioinformatics.babraham.ac.uk/projects/fastqc/).

At this time, low quality bases (> 20) or adapter sequences were trimmed using TrimGalore (Version 0.5.0) with “-q 20 -phred33 -rrbs -paired -non_directional” options. Then, alignment to the reference genome of the trimmed reads were performed with Bismark (Version 0.20.0) with the “-non_directional” option.

The Bismark-resultant .sam files were converted into .bam files with Samtools (Version 1.9). And, methylated positions were called with the processBismarkAln of methylKit (Version 1.10.0). At this time, the number of methylated cytosines in 1000 bases of non-overlapping windows across the whole genome was estimeted by using the tileMethylCounts function from the methylKit package. Then, normalization was conducted with the normalizeCoverage function in methylKit. Bases having below 10 read coverage and bases with more than 99.9th percentile of coverage in each sample (likely PCR artifacts with abnormally high coverage) were removed using the filterByCoverage function of methylKit. All the samples were then merged with using the unite function in methylKit with the destrand = FALSE option.t-SNE plots of fold changes of each β-value compared with the control in the chemical treatment samples were created using iDEP.96 software.

Distributions of the genome area of differential methylated site of each chemical and DNA methylation rate at CpG sites around the locus of *JAG1*, *LIFR*, *CCND2*, and *RB1* genes were created by using EZR (Supplementary Table S12)^57^.

### Statistical analyses

In Figs. 1D,E and 4A, statistical significance was determined using one-way ANOVA test, followed by Tukey’s multiple comparison test for determining the statistical significance between two among all groups. In Figs. 2B and 3C, statistical significance was determined using Kruskal–Wallis test, and Steel–Dwass test was used to determine the statistical significance between two among all groups. All statistical analyses were two sided and *P*-values of < 0.05 and < 0.01 were considered statistically significant. All statistical analyses were performed using EZR^57^. All *P*-values calculated are listed in Supplementary Tables S13–S16.

## Supplementary Information

Supplementary Tables.

Supplementary Figures.

Supplementary Information.

## Data Availability

The DNA methylation array and transcriptome data are MIAME compliant, and the raw data have been deposited in the Gene Expression Omnibus (www.ncbi.nlm.nih.gov/geo, series of data, GSE180454; DNA methylation array data, GSE180453; transcriptome data, GSE180452; RRBS data, GSE180454, GSE228658). Further information for resources, raw data, and reagents are available from the corresponding author on reasonable request.
