# Supplementary material for: iGEM as a human iPS cell-based global epigenetic modulation detection assay provides throughput characterization of chemicals affecting DNA methylation
Source: Sci Rep. 2023 Apr 24;13:6663. doi: 10.1038/s41598-023-33729-4 (PMC10125974; doi:10.1038/s41598-023-33729-4)
Supplement: Supplementary file 2 — Supplementary Figures. [file 41598_2023_33729_MOESM2_ESM.docx]

**
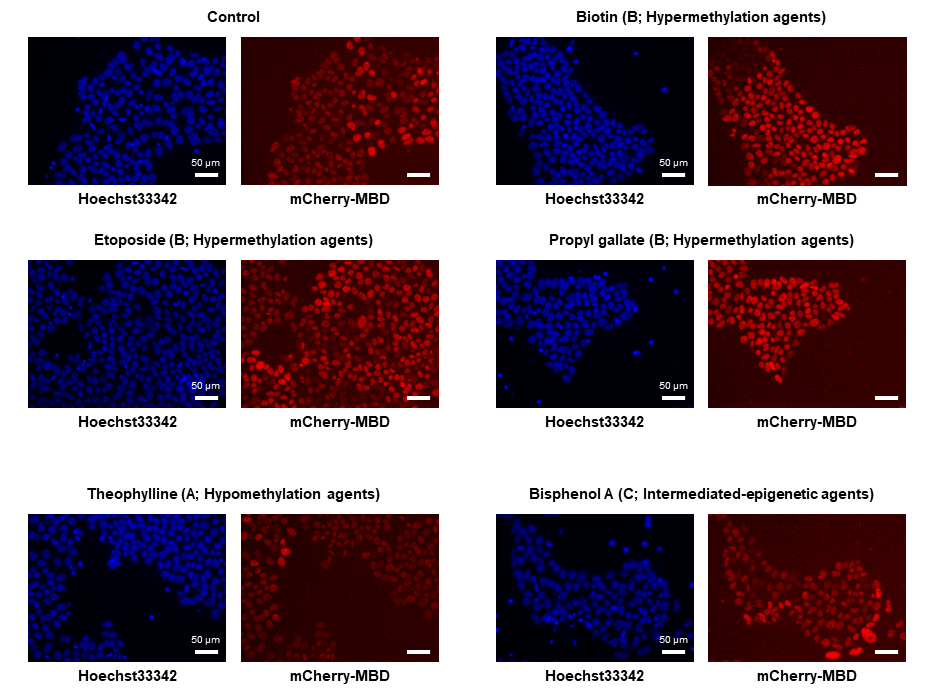
**

Supplementary Figure 1. Representative microscopic images of mCherry-MBD-nls human iPSCs.

Images of cells treated with 0.1 % DMSO (Control), 1 × 10^−6^ M biotin (Biotin), 1 × 10^−9^ M etoposide (Etoposide), and 1 × 10^−6^ M propyl gallate (Propyl gallate), 1 × 10^−6^ M theophylline, and 3 × 10^−8^ M bisphenol A (Bisphenol A) for 48 h were taken using IN Cell analyzer 1000. Blue; Hoechst33342, Red; mCherry-MBD.


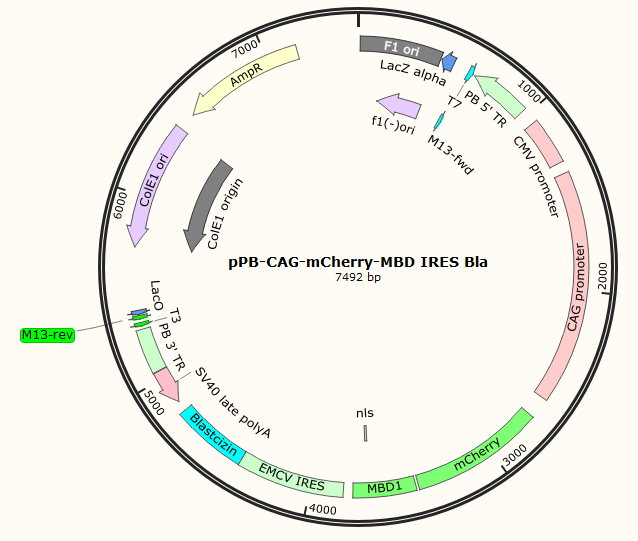


Supplementary Figure 2. The sequence map of the mCherry-MBD-nls construct.
